# CD163 versus CD68 in tumor associated macrophages of classical hodgkin lymphoma

**DOI:** 10.1186/1746-1596-7-12

**Published:** 2012-01-30

**Authors:** Jonathan A Harris, Salvia Jain, Qinghu Ren, Alirezah Zarineh, Cynthia Liu, Sherif Ibrahim

**Affiliations:** 1 Department of Pathology, New York University Langone Medical Center, 560 1st Ave, New York, New York, 10016, USA; 2 Department of Medicine, Hematology/Oncology, New York University Langone Medical Center, 560 1st Ave, New York, New York, 10016, USA

## Abstract

Classical Hodgkin lymphoma (CHL) is a B-cell lymphoproliferative disorder with a relatively good prognosis. A small but significant percentage of patients, however, will respond poorly to therapy. A recent gene expression profiling study has identified a macrophage signature which has been correlated with primary treatment failure, and immunohistochemical tissue microarray for CD68 was shown to reflect the gene signature as a potentially clinically useful marker to predict adverse prognosis.

We examined 44 cases of CHL, mostly nodular sclerosis subtype, in which the immunohistochemical stains for the histiocytic markers CD68 and CD163 were performed. The staining intensity was graded for each stain (< 5, 5-25, and > 25 percent of cells positive in the Hodgkin cell (HC) rich nodules) and background staining characteristics were recorded.

CD163 staining was lower than CD68 in HC rich nodules, with lower background staining (p 0.03). There was no significant difference between either CD68 or CD163 and disease recurrence in a subset (N = 41) of cases.

In conclusion, we demonstrate that CD163 staining is lower than CD68, with less non-specific staining of background inflammatory cells and Hodgkin cells, therefore is a better marker for tumor associated macrophages. However, we did not identify a correlation between staining for CD68 or CD163 and recurrence of disease.

**Virtual slides:**

The virtual slide(s) for this article can be found here:

http://www.diagnosticpathology.diagnomx.eu/vs/1460518258831620

## Introduction

Classical Hodgkin lymphoma is a B cell lymphoma with a relatively good prognosis. However, approximately 20% of patients will be refractory to primary treatment or relapse after remission [1]. The cellular microenvironment has been extensively studied and plays an important part in the pathogenesis of Hodgkin lymphoma. Tissue microarray studies have proven useful in the study of Hodgkin lymphoma [2-4], in which the neoplastic cells are relatively few compared with the highly cellular inflammatory and stromal background. Several studies have used gene expression profiles to study the microenvironment in classical Hodgkin lymphoma [5-7]

Tumor associated macrophages have been associated with disease status in non-hematologic malignancies [8]. A macrophage gene profile in classical Hodgkin lymphoma was identified in two studies, and was associated with unfavorable outcome [2,6]. The former study also used tissue microarray immunohistochemical staining for the monocyte/macrophage marker, CD68 on an independent cohort of patients and found high numbers of tumor associated macrophages were associated with shortened progression free survival and increased likelihood of relapse post autologous stem cell transplant. In addition, this study found a low CD68 score was associated with 100% disease-specific survival in patients with limited stage disease (stage I and IIa). Assuming these immunohistochemical findings can be reproduced, this may indicate the necessity of a practical approach to enumerating macrophages in the everyday practice of pathology.

CD68 (Kp-1) is a glycoprotein used as a monocyte/macrophage marker but is relatively non-specific. It also can stain myeloid cells, dendritic cells, fibroblasts, Langerhans cells and others. CD163 is a member of the scavenger receptor family and is specific for the monocyte/macrophage lineage [9,10]. We tested 44 cases of classical Hodgkin lymphoma for antibodies to both CD68 and CD163 to determine if CD163 may be a better macrophage marker to enumerate tumor associated macrophages in classical Hodgkin lymphoma.

In addition, we concurrently performed chart review on a subset of 41 patients to compare level of staining with disease recurrence following treatment.

## Materials and methods

We searched the pathology database at our institution for cases of classical Hodgkin lymphoma diagnosed between January 2000 and August 2010. Cases were selected based on available blocks with adequate tissue (~1 cm^2^). Adequate diagnostic material was found for 44 cases, most of which were nodular sclerosis subtype (Table 1). This study was approved by the institutional review board at the NYU School of Medicine.

**Table 1:** Clinical information and analysis results

Demographics			Clinical Characteristics		Immunohistochemical Analysis^2^		
Patient	Age	Sex	Stage	Tumor size (cm)^1^	CD68	CD163	p value
1	21	F	IIA	6	2	1	0.01
2	73	M	IIIB	3	2	1	
3	37	M	IIA	2	3	2	
4	59	F	IIA	4.3	3	3	
5	42	F	IIA	4	3	3	
6	94	M	IIA	2.2	3	1	
7	20	F	IIIA	> 10	2	1	
8	19	F	IIA	> 10	3	3	
9	51	M	IVA	> 10	3	3	
10	28	M	IIA	2.5	2	1	
11	33	F	IVB	3	2	2	
12	62	F	IIB	2	2	2	
13	15	F	IIIA	> 10	2	2	
14	22	M	IIA	2	1	1	
15	23	F	IIB	> 10	1	1	
16	33	M	IA	2.8	2	2	
17	48	M	IB	3	2	2	
18	17	F	IVB	5	2	2	
19	25	F	IIB	17	1	1	
20	52	M	IVB	4	3	3	
21	45	M	IVB	11	2	1	
22	20	M	IIB	2	3	3	
23	64	M	IVB	5.5	1	1	
24	23	M	IIA	4	3	3	
25	14	M	IVB	9.4	2	2	
26	14	F	IVB	3	1	1	
27	35	M	IIA	1.8	2	1	
28	54	M	IIIB	4.9	2	1	
29	40	M	IV	2.2	3	1	
30	21	F	IA	10	3	3	
31	34	F	IA	6.3	2	2	
32	17	F	IVB	5.1	2	3	
33	22	F	IIB	7.5	2	2	
34	66	F	IIIA	1.7	2	2	
35	26	F	IVA	1.6	1	1	
36	24	M	IV	6.3	2	2	
37	35	M	IIA	1.5	3	3	
38	23	F	IIA	2.5	3	3	
39	26	M	IVB	2.3	2	2	
40	18	F	IIIA	6	1	2	
41	49	M	IA	9	2	2	
42	70	F	IVB	2.4	2	1	
43	21	M	IIA	3	1	1	
44	38	M	IA	4	3	3	

^1 ^The tumor size was calculated as the largest diameter of the largest involved LN

^2 ^Score of 1 = 0-5%, 2 = 5-25%, and 3 = > 25% staining of total cells in HRS rich areas

We performed immunohistochemical stains using CD68, Clone KP-1, and CD163, Clone MRQ-26, (Ventana Medical Systems, Tucson AZ) on representative formalin fixed, paraffin embedded tissue blocks from each case. In brief, sections were deparaffinized in xylene (3 changes), rehydrated through graded alcohols (3 changes 100% ethanol, 3 changes 95% ethanol) and rinsed in distilled water. Heat induced epitope retrieval was performed in a 1200-Watt microwave oven at 90% power using 0.01 M Citrate buffer pH 6.0 for 5 and 20 minutes respectively. Sections were allowed to cool for 30 minutes and then rinsed in distilled water. Antibody incubations and detection were carried out at 37°C on a NEXes instrument (Ventana Medical Systems Tucson, AZ) using Ventana's reagent buffer and detection kits unless otherwise noted. Endogenous peroxidase activity was blocked with hydrogen peroxide. Both antibodies were applied neat and incubated for 30 minutes. Primary antibody was detected using a biotinylated goat anti-mouse followed by application of streptavidin-horseradish-peroxidase conjugate. The complex was visualized with 3,3 diaminobenzidene and enhanced with copper sulfate. Slides were washed in distilled water, counterstained with hematoxylin, dehydrated and mounted with permanent media. Appropriate positive and negative controls were included with the study sections.

Stains were compared with corresponding H&E stained sections. The level of CD68 and CD163 staining was recorded by two independent investigators (JH, QR) and graded with respect to the relative percentage of tumor associated macrophages in HRS (Hodgkin Reed-Sternberg cells) rich areas. The few discrepant cases were reviewed together until agreement was reached. The percentage was recorded and graded as: 1 if less than 5%, 2 if 5-25%, and 3 if greater than 25% of the total cells present in HRS rich nodules were positive for CD68 or CD163.

Statistical analysis was performed using a Student's t-test to compare the staining between CD68 and CD163.

Independent chart review was simultaneously performed on 41 cases to determine the clinical outcome since the original diagnosis. Disease stage, time to disease recurrence, and treatment regimen were recorded, along with lines of treatment (chemotherapy versus autologous stem cell transplant, etc). Disease recurrence occurred in 9 patients, 4 with limited stage disease (stage I or IIa).

For comparison study, outcome was good if disease free and poor if disease recurred. Both CD68 and CD163 were compared to both outcome groups using a Fisher Exact Probability Test with Freeman-Halton 2 × 3 extension to compare the groups of disease free and disease recurrence with the three staining levels.

## Results

**Figure 1:**
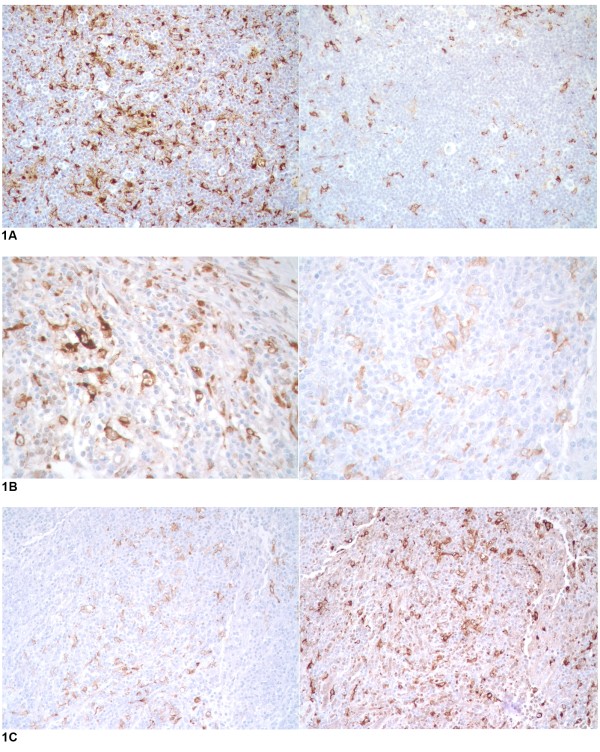
**Immunohistochemical Results, a: CD163 (right) showed lower staining levels than CD68 in HRS rich areas**. b: CD163 (right) staining pattern shows cleaner background than CD68, with less non-specific staining. c: CD68 showed more variability in staining within the same tissue, with grade 3 staining (right) in some nodules and grade 1 staining (left) in adjacent HRS rich nodules.

**Figure 2:**
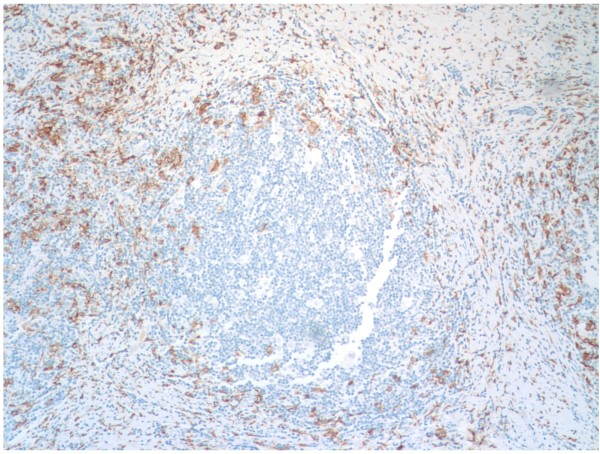
**CD163 staining is higher in the sclerotic bands than in the HRS rich nodules**.

After analyzing 44 cases of classical Hodgkin lymphoma, CD163 showed lower staining levels than CD68 in HRS rich areas. (p 0.01)(Figure 1a). CD163 staining pattern showed a more clean background than CD68, with less non-specific staining of Hodgkin Reed-Sternberg cells and other inflammatory elements (Figure 1b). CD68 showed more variability in staining within the same tissue, with grade 3 staining in some nodules and grade 1 staining in adjacent HRS rich nodules (Figure 1c). CD163 staining pattern was characteristically higher in the sclerotic bands as compared with the HRS rich nodules (Figure 2). CD163 showed more consistent staining, with only one case showing more CD163 than CD68. Two cases showed significantly more staining for CD68 than with CD163 (grade 3 compared to grade 1) with all other discrepant cases showing only a one grade difference. CD68 stained HRS cells more frequently than did CD163.

Analysis of the subset of 41 patients revealed no statistical difference among the three grading groups for CD68 or CD163 and disease recurrence (p 0.66 and p 0.70 for CD68 and CD163 respectively). In the two cases with significantly more CD68 than CD163, and the one case with more CD163 than CD68, there was no disease recurrence.

## Discussion

Tumor associated macrophages play an important role in classical Hodgkin lymphoma, with reported gene signatures associated with adverse outcome following primary treatment [2]. We used immunohistochemical staining for the macrophage/monocyte markers CD68 and CD163 to directly compare the staining characteristics in 44 cases of classical Hodgkin lymphoma. While CD68 is a marker for cells of the monocyte/macrophage lineage, it is relatively nonspecific, with reported staining in neoplasms such as carcinoma, melanoma, angiosarcoma, lymphoma, and schwannoma [10]. There is also nonspecific staining in fibroblasts and inflammatory cells [9], a finding we demonstrated in this study. CD163 has been shown to be highly specific for tumors of the monocyte/macrophage lineage [10].

CD163 showed lower staining than CD68 in HRS rich areas, with lower background staining and less staining of HRS cells. In cases with extremely high levels of CD68 and CD163, we did find occasional HRS cells staining for CD163, but in much fewer numbers than were staining for CD68. One study reported that CD163 may be down-regulated by the T-helper immune response in synovial tissues [9], suggesting that it may be underrepresented in tumor associated macrophages. Although we did show fewer cells staining for CD163 in the HRS rich nodules, there were several cases with abundant CD163 staining, which does not support this finding.

Subjectivity is inherent in the enumeration of cells in histologic sections. Immunohistochemical stains which non-specifically highlight background cells and stromal elements complicate the matter further. In addition, variability of staining from one area of the slide to the next can make an accurate determination even more challenging. Tissue microarray is a powerful tool but cannot completely address the variability of staining from one area of the tissue to another, even in paired core analysis. Ideally, an immunohistochemical stain can specifically highlight the cells of interest, and enumeration can be accurately reproduced by multiple pathologists. In the case of classical Hodgkin lymphoma, there is a myriad of inflammatory and stromal elements surrounding the relatively few neoplastic cells, and high background staining can complicate matters when using the non-specific immunostain CD68. As most cases of classical Hodgkin lymphoma are nodular sclerosis type, the HRS rich areas are usually confined to the centers of the nodules, simplifying the evaluation of immunohistochemical markers by limiting the grading to those nodules. CD163 staining characteristically shows a honeycomb pattern on low power, with relatively few cells in the nodules staining and more cells in the surrounding fibrotic stroma. We do acknowledge there is generally not an ideal marker for the tissue identification of subsets of macrophages, however, as compared with the commonly used antibody to CD68 (KP-1), CD163 appears to be a better marker for enumeration of tumor associated macrophages classical Hodgkin lymphoma.

Of note, we did not find a correlation between staining level and disease recurrence in the subgroup of 41 patients. Of the 9 patients with disease recurrence, 4 patients had limited stage disease. One patient with recurrent disease had < 5% staining (grade 1) for both CD68 and CD163. Our findings support those of a recent study by Azambuja, et al, which found no correlation between the numbers of CD68 and CD163 positive cells and either progression free survival or disease specific survival in 265 cases of uniformly treated classical Hodgkin lymphoma [11]. Although they did not directly compare CD163 to CD68 statistically, they noted that CD163 is an easier stain to read as there is less background than CD68.

In conclusion, we determined that CD163 is a better marker than CD68 for the enumeration of tumor associated macrophages in the everyday practice of surgical pathology. Our study, together with the larger study by Azambuja, et al, shows evidence that there is no difference between histologically enumerated macrophages and clinical outcome in cases of classical Hodgkin lymphoma. Together, these findings do not support the previously published findings of Steidl, et al [2]. Additional work may be necessary to determine the validity of routine staining for tumor associated macrophages.

## Competing interests

The authors declare that they have no competing interests.

## Authors' contributions

JAH participated in the design of the study, performed the histologic/immunohistochemical evaluation and statistical analysis, and drafted the manuscript. SI and CL conceived the study and participated in the design and coordination. SJ collected all of the clinical data for the study and participated in drafting the manuscript. AZ and QR participated in histologic/immunohistochemical evaluation, data analysis, and drafting the manuscript. All authors have read and approved the final manuscript.
